# Supplementary material for: Per-allele disease and complex trait effect sizes are predominantly African MAF-dependent in European populations
Source: medRxiv. 2026 Jan 19:2025.12.31.25343290. Preprint. [Version 2] doi: 10.64898/2025.12.31.25343290 (PMC12838302; doi:10.64898/2025.12.31.25343290)
Supplement: Supplement 2 [file NIHPP2025.12.31.25343290v2-supplement-2.pdf]

medRxiv preprint doi: https://doi.org/10.64898/2025.12.31.25343290; this version posted January 19, 2026. The copyright holder for this preprint (which was not certified by peer review) is the author/funder, who has granted medRxiv a license to display the preprint in perpetuity.
It is made available under a CC-BY 4.0 International license .

Supplementary Tables:

Supplementary Table 1: Overview of 50 diseases/complex traits analyzed. We report the SNP heritability (h^2) as estimated by LDSC and GWAS sample size.
(see .xlsx file)

Supplementary Table 2: The proportion of non-synonymous SNPs in a grid of bivariate African and European MAF bins. We report the proportion of non-synonymous SNPs, the proportion of synonymous SNPs, the number of SNPs, and the Wald standard error on the proportion of non-synonymous SNPs.
(see .xlsx file)

Supplementary Table 3: The proportion of non-synonymous SNPs in African MAF deciles stratified by European MAF bins. We report, for each decile, the African MAF, the proportion of non-synonymous variants, 95% confidence intervals on the proportion of non-synonymous variants, and the European MAF bin. We restricted to variants with $p_A \geq 0.002$
(see .xlsx file)

Supplementary Table 4: Predictions from logistic regressions of SNP non-synonymous status on African MAF stratified by European MAF bins. We report the predicted proportion of non-synonymous variants (and 95% confidence intervals) for a grid of 30,000 African MAF values from 0.002 - 0.5. In the regression African MAF was log transformed. We restricted to variants with $p_A \geq 0.002$.
(see .xlsx file)

Supplementary Table 5: The proportion of non-synonymous SNPs in European MAF deciles stratified by African MAF bins. We report, for each decile, the European MAF, the proportion of non-synonymous variants, 95% confidence intervals on the proportion of non-synonymous variants, and the African MAF bin. We restricted to variants with $p_E \geq 0.002$
(see .xlsx file)

Supplementary Table 6: Predictions from logistic regressions of SNP non-synonymous status on European MAF stratified by African MAF bins. We report the predicted proportion of non-synonymous variants and 95% confidence intervals for a grid of 30,000 European MAF values from 0.002 - 0.5. In the regression European MAF was log transformed. We restricted to variants with $p_E \geq 0.002$.
(see .xlsx file)

Supplementary Table 7: Best-fit parameters for a logistic regression of non-synonymous status on log(p_{mix}). We report the log-likelihood, γ estimate, γ standard error, and 95% confidence interval on γ across 101 values of w from 0 to 1.
(see .xlsx file)

medRxiv preprint doi: https://doi.org/10.64898/2025.12.31.25343290; this version posted January 19, 2026. The copyright holder for this preprint (which was not certified by peer review) is the author/funder, who has granted medRxiv a license to display the preprint in perpetuity.
It is made available under a CC-BY 4.0 International license .

Supplementary Table 8: Per-allele effect size variance across 50 diseases/traits for a grid of bivariate African and European MAF bins. We report point estimates and standard errors of the meta-analyzed scaled mean effect size variance for each bin.
(see .xlsx file)

Supplementary Table 9: Per-allele effect size variance across 50 diseases/traits as a function of MAF quintiles, stratified by African MAF. We report, for each MAF quintile, the mean MAF, point estimate of the meta-analyzed scaled mean effect size variance, standard error of the meta-analyzed scaled mean effect size variance, and 95% confidence interval on the meta-analyzed scaled mean effect size variance.
(see .xlsx file)

Supplementary Table 10: Results of fitting the α_{mix} model across 50 diseases/traits. We report point estimates and standard errors on α_{mix} and point estimates and standard errors of the loss function across 101 values of *w* from 0 to 1.
(see .xlsx file)

Supplementary Table 11: Results of fitting the α_{mix} model separately for 50 diseases/traits. We report point estimates and standard errors on *w*, α_{mix}, and the loss function for each of 50 diseases/traits.
(see .xlsx file)

Supplementary Table 12: Results of fitting the α_{mix} model in simulations. We report point estimates and standard errors on the mean estimate of *w* and α_{mix}.
(see .xlsx file)

medRxiv preprint doi: https://doi.org/10.64898/2025.12.31.25343290; this version posted January 19, 2026. The copyright holder for this preprint (which was not certified by peer review) is the author/funder, who has granted medRxiv a license to display the preprint in perpetuity.
It is made available under a CC-BY 4.0 International license .

Supplementary Figures:

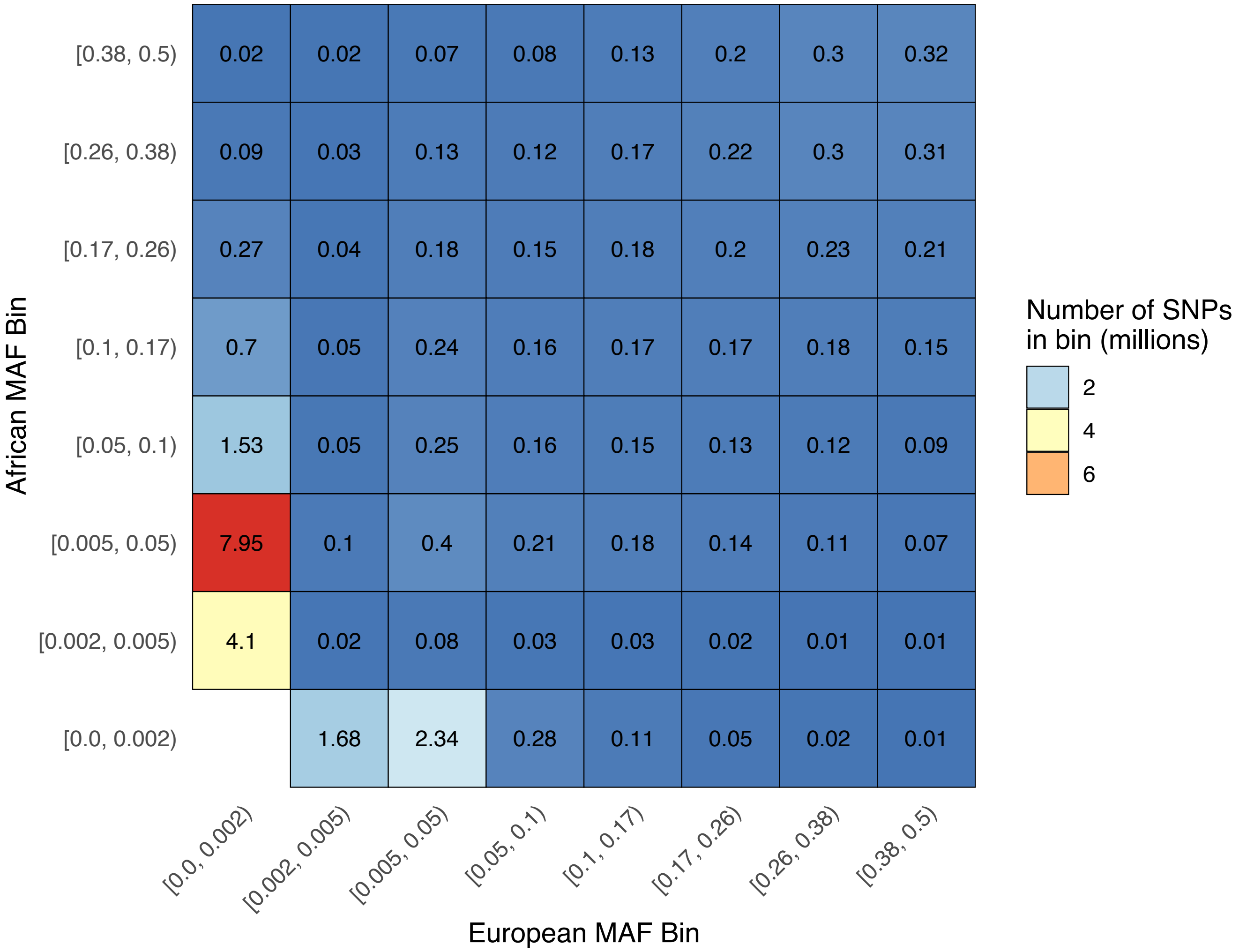

Supplementary Figure 1: Number of SNPs in each AoU MAF stratum. We report the number of SNPs contained in each grid element given in Figure 1a. Each grid element contains SNPs with unadmixed African and European MAF that fall in a pair of MAF intervals. A justification for MAF interval boundaries is given in Methods.

medRxiv preprint doi: https://doi.org/10.64898/2025.12.31.25343290; this version posted January 19, 2026. The copyright holder for this preprint (which was not certified by peer review) is the author/funder, who has granted medRxiv a license to display the preprint in perpetuity.
It is made available under a CC-BY 4.0 International license .

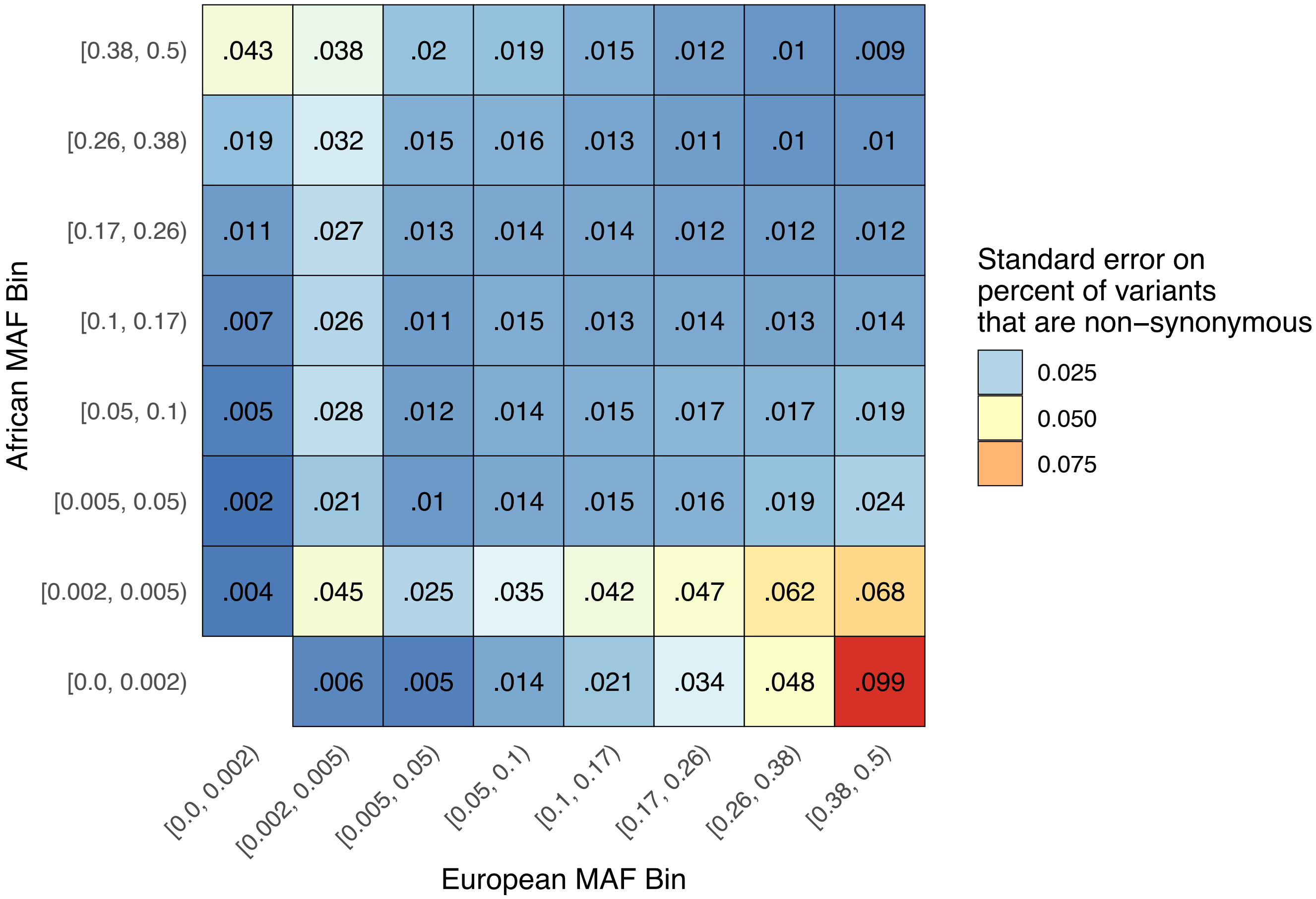

Supplementary Figure 2: Standard error on the proportion of non-synonymous SNPs in each MAF stratum. We report the binomial proportion standard error for each grid element given in Figure 1a. Each grid element contains SNPs with unadmixed African and European MAF that fall in a pair of MAF intervals. A justification for MAF interval boundaries is given in Methods.

medRxiv preprint doi: https://doi.org/10.64898/2025.12.31.25343290; this version posted January 19, 2026. The copyright holder for this preprint (which was not certified by peer review) is the author/funder, who has granted medRxiv a license to display the preprint in perpetuity.
It is made available under a CC-BY 4.0 International license .

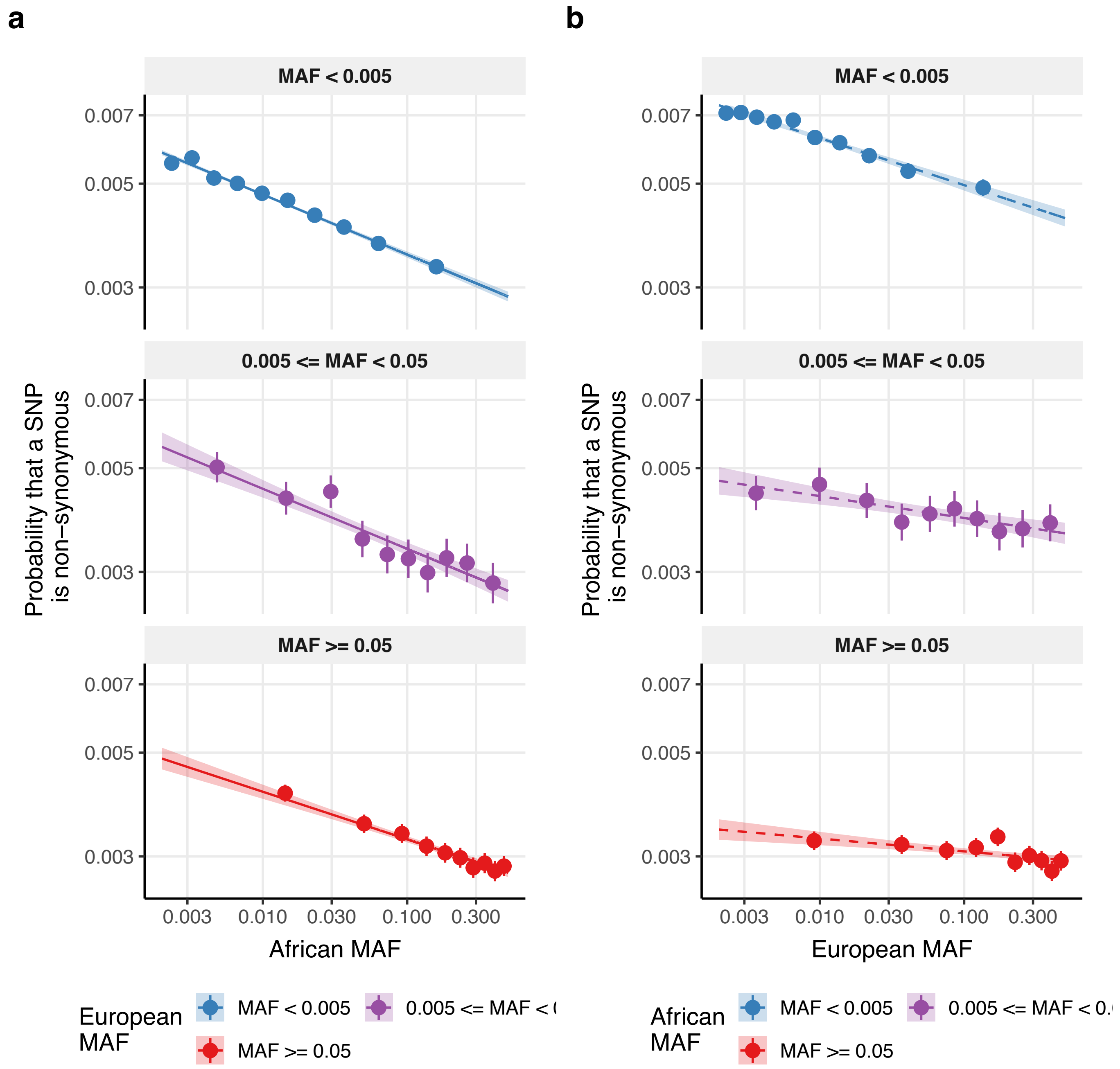

Supplementary Figure 3: Logistic regression for non-synonymous status as a function of unadmixed African and European minor allele frequencies separated by MAF category. We report **(a)** the probability that a SNP is non-synonymous as a function of African MAF after stratifying by European MAF categories (for SNPs with $p_A \geq 0.002$) as predicted by logistic regression, and **(b)** the probability that a SNP is non-synonymous as a function of European MAF after stratifying by African MAF categories (for SNPs with $p_E \geq 0.002$) as predicted by logistic regression. Error bars denote 95% Agresti-Coull confidence intervals. Shaded regions in (b) and (c) denote 95% confidence intervals on the mean estimate. Logistic regression fits match non-parametric MAF deciles remarkably well, indicating that our conclusions are unlikely to be impacted by model misspecification.

medRxiv preprint doi: https://doi.org/10.64898/2025.12.31.25343290; this version posted January 19, 2026. The copyright holder for this preprint (which was not certified by peer review) is the author/funder, who has granted medRxiv a license to display the preprint in perpetuity.
It is made available under a CC-BY 4.0 International license .

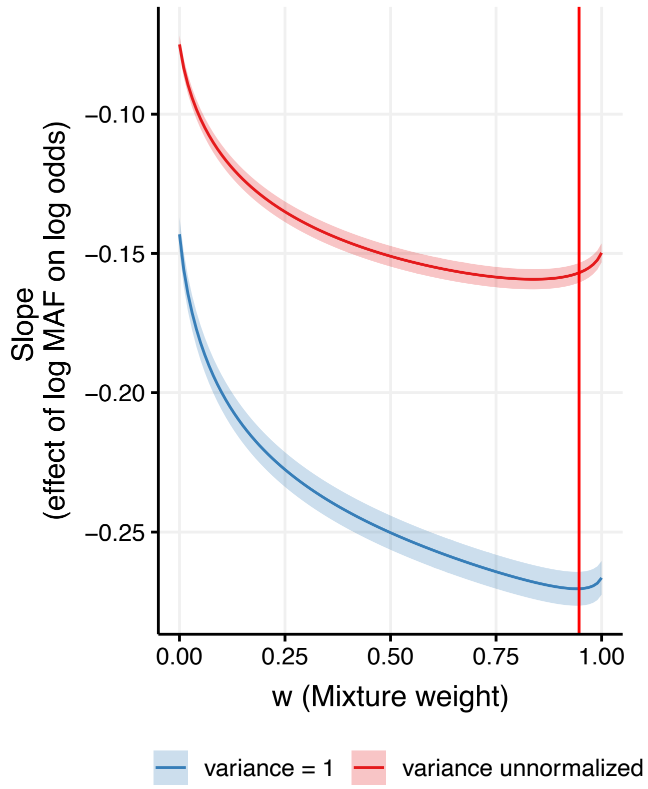

Supplementary Figure 4: Best fit parameter for a logistic regression of non-synonymous status on log(p_{mix}) while scaling log(p_{mix}) to have unit variance. We report the regression slope (γ) for values of w between 0 and 1. The blue curve denote results for a logistic regression on log(p_{mix}) while scaling log(p_{mix}). The red curve denotes results for a logistic regression on log(p_{mix}) without scaling log(p_{mix}). The vertical red line denotes the MLE of w=0.95 (the MLE of w is not affected by scaling log(p_{mix})). With scaling, w=0.94 maximizes the absolute slope. Without scaling, w=0.84 maximizes the absolute slope.

medRxiv preprint doi: https://doi.org/10.64898/2025.12.31.25343290; this version posted January 19, 2026. The copyright holder for this preprint (which was not certified by peer review) is the author/funder, who has granted medRxiv a license to display the preprint in perpetuity.
It is made available under a CC-BY 4.0 International license .

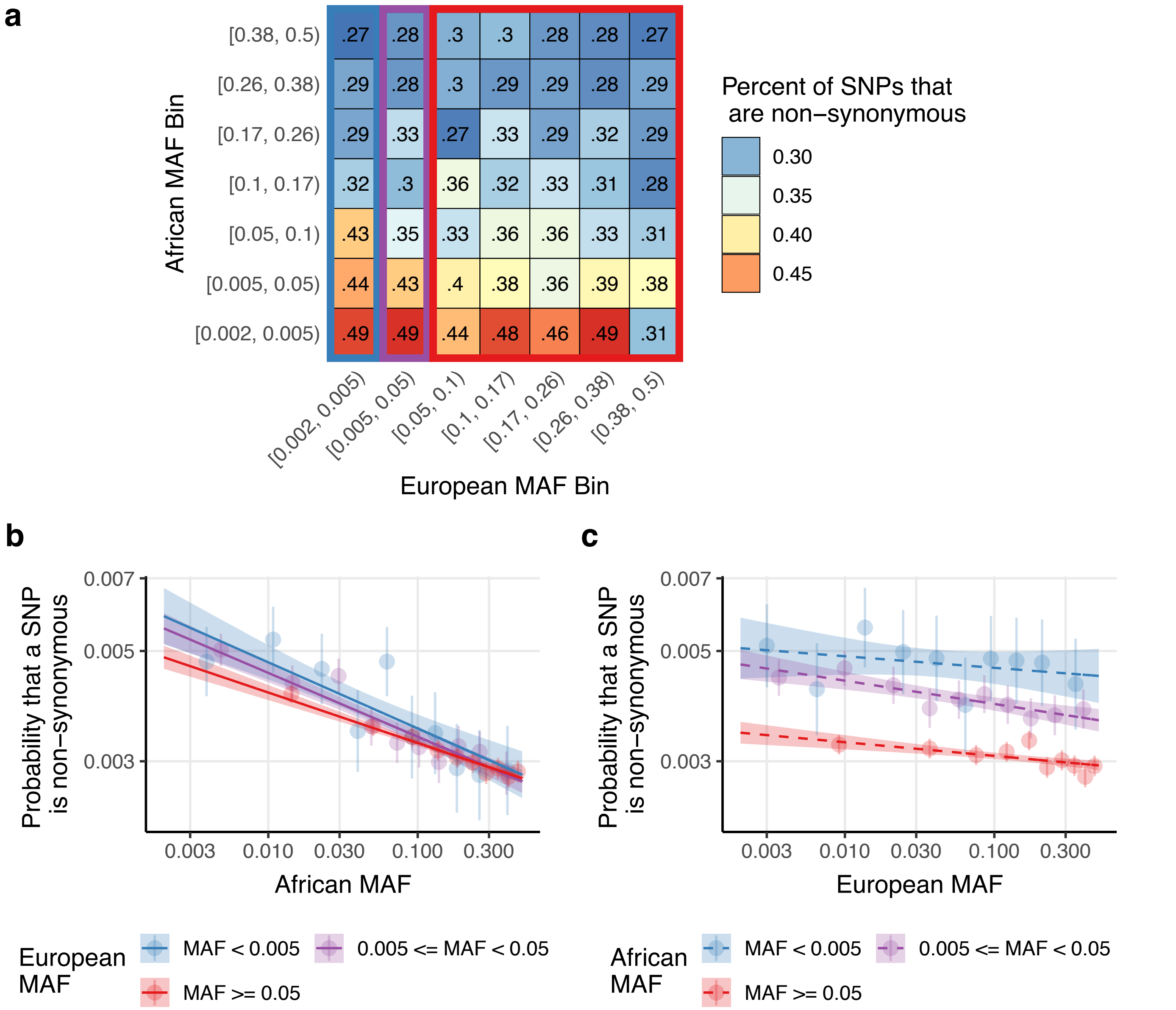

Supplementary Figure 5: The proportion of non-synonymous SNPs as function of unadmixed African and European minor allele frequencies, restricted to variants with $p_A \geq 0.002$ and $p_E \geq 0.002$: We report **(a)** the percentage of SNPs that are non-synonymous in bivariate bins defined by unadmixed African MAF (rows) and European MAF (columns), **(b)** the probability that a SNP is non-synonymous as a function of African MAF after stratifying by European MAF categories (for SNPs with $p_A \geq 0.002$ and $p_E \geq 0.002$) as predicted by logistic regression, and **(c)** the probability that a SNP is non-synonymous as a function of European MAF after stratifying by African MAF categories (for SNPs with $p_E \geq 0.002$ and $p_A \geq 0.002$) as predicted by logistic regression. The blue, purple, and red rectangles in (a) correspond to the SNPs included in the blue, purple, and red lines in (b). The choice of MAF bin boundaries is discussed in Methods. Shaded regions in (b) and (c) denote 95% confidence intervals on the mean estimate. Error bars denote 95% Agresti-Coull confidence intervals.

medRxiv preprint doi: https://doi.org/10.64898/2025.12.31.25343290; this version posted January 19, 2026. The copyright holder for this preprint (which was not certified by peer review) is the author/funder, who has granted medRxiv a license to display the preprint in perpetuity.
It is made available under a CC-BY 4.0 International license .

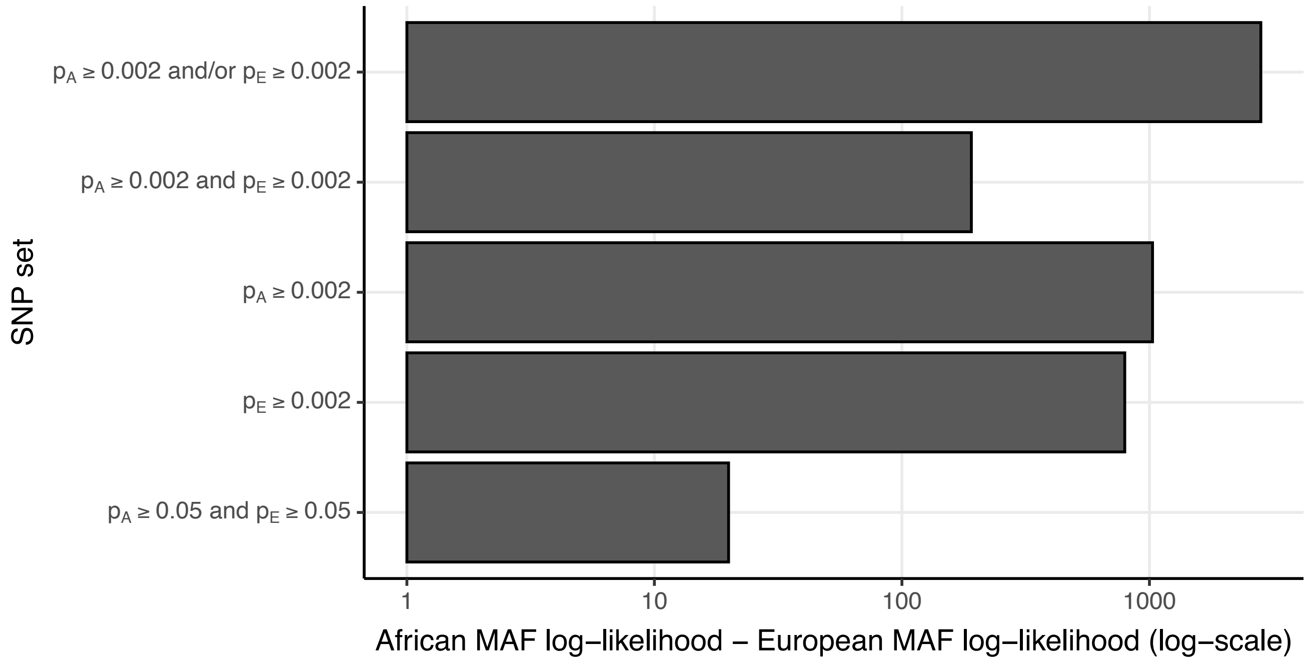

Supplementary Figure 6: Difference in log-likelihood for a logistic regression of non-synonymous status on African MAF vs. a logistic regression of non-synonymous status on European MAF. We report the log-likelihood for a logistic regression of PNS on log(p_A) minus the log-likelihood for a logistic regression of PNS on log(p_E), while varying the SNP set. All log-likelihood differences were significant via Vuong's test. For each SNP set, the exact same set of SNPs were included in the African MAF-only regression and the European MAF-only regression. For all SNPs, we thresholded MAF values at 0.002 after filtering for SNP set. That is, if $p_A < 0.002$, p_A was set to 0.002, and if $p_E < 0.002$, p_E was set to 0.002 (after restricting to the set of SNPs to be included in each SNP set).

medRxiv preprint doi: https://doi.org/10.64898/2025.12.31.25343290; this version posted January 19, 2026. The copyright holder for this preprint (which was not certified by peer review) is the author/funder, who has granted medRxiv a license to display the preprint in perpetuity.
It is made available under a CC-BY 4.0 International license .

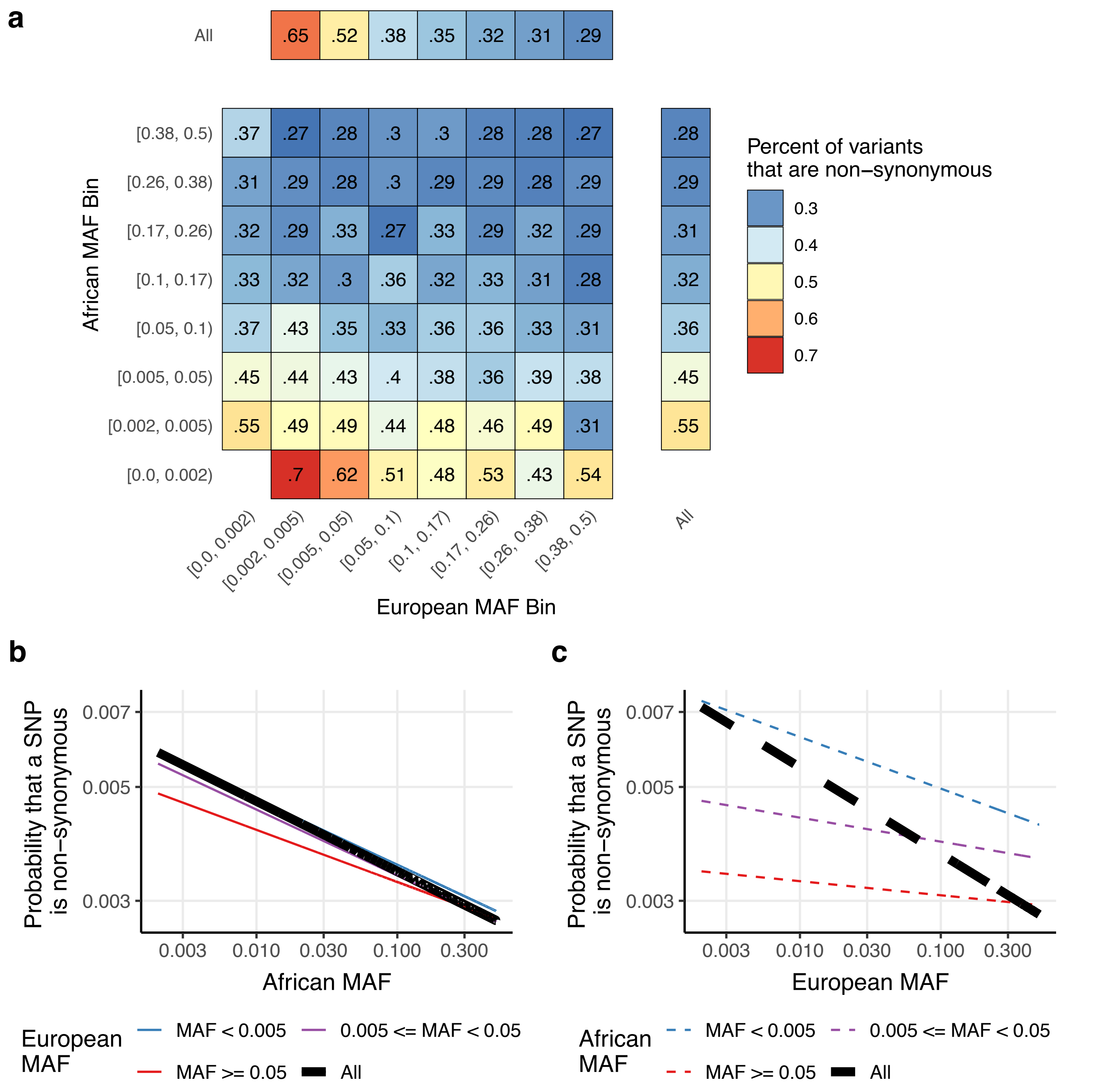

Supplementary Figure 7: The proportion of non-synonymous SNPs as function of unadmixed African and European minor allele frequencies, highlighting the marginal effects of African MAF for SNPs with $p_A \geq 0.002$ and European SNPs with $p_E \geq 0.002$: We report **(a)** the percentage of SNPs that are non-synonymous in univariate bins defined by unadmixed African MAF (rightmost column) and European MAF (top row) and bivariate bins defined by unadmixed African MAF (other rows) and European MAF (other columns), **(b)** the probability that a SNP is non-synonymous as a function of African MAF marginally (black) and after stratifying by European MAF categories (colored lines) (for SNPs with $p_A \geq 0.002$) as predicted by logistic regression, and **(c)** the probability that a SNP is non-synonymous as a function of European MAF marginally (black) and after stratifying by African MAF categories (colored lines) (for SNPs with $p_E \geq 0.002$) as predicted by logistic regression.

medRxiv preprint doi: https://doi.org/10.64898/2025.12.31.25343290; this version posted January 19, 2026. The copyright holder for this preprint (which was not certified by peer review) is the author/funder, who has granted medRxiv a license to display the preprint in perpetuity.
It is made available under a CC-BY 4.0 International license .

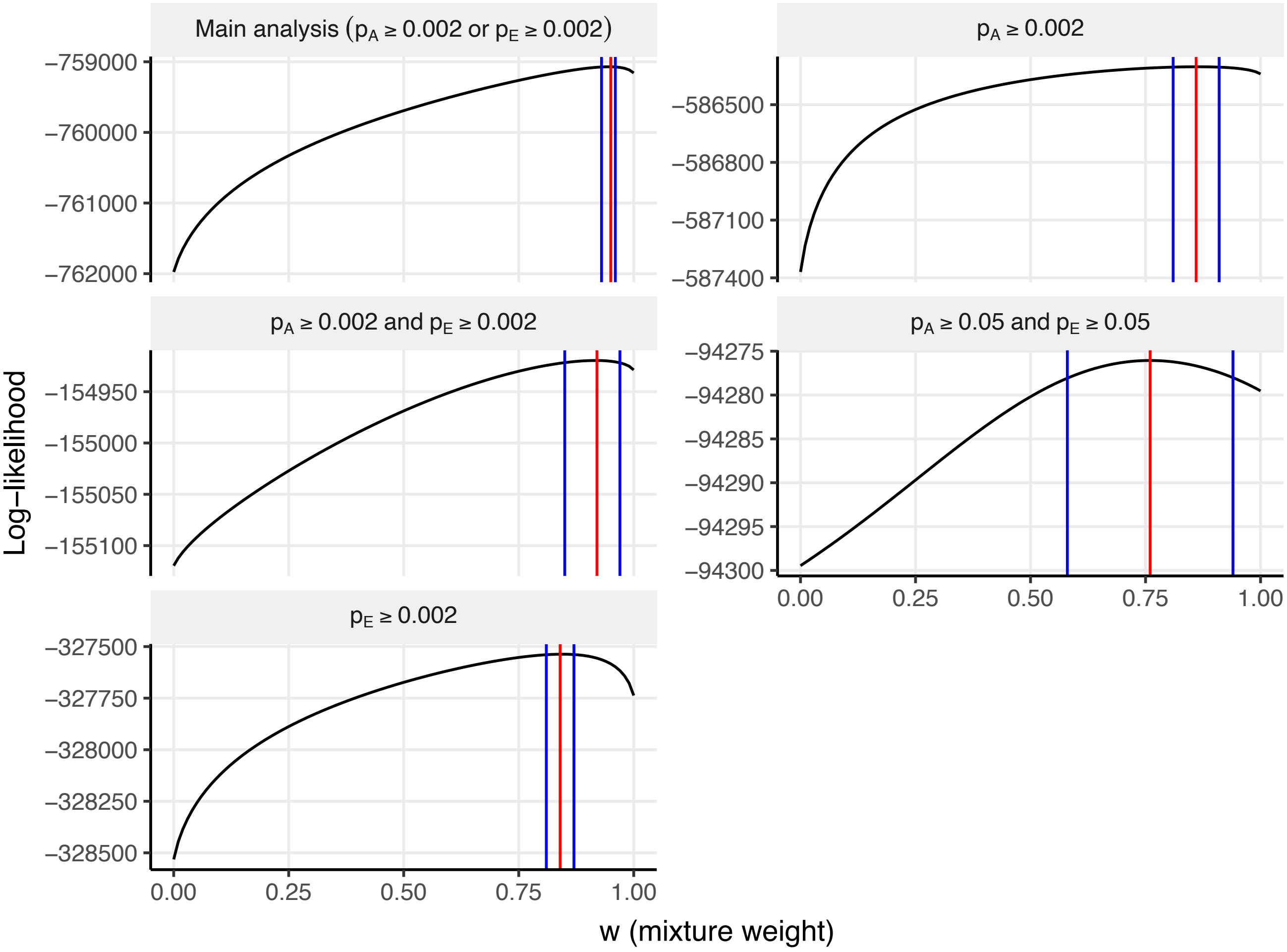

Supplementary Figure 8: Log-likelihood for a logistic regression of non-synonymous status on log(p_{mix}), varying the set of SNPs analyzed. We report the profile log-likelihood with respect to *w*, treating the regression intercept and slope as nuisance parameters. Each curve corresponds to a distinct set of SNPs included while fitting the regression. Red lines denotes the MLE for *w*. Blue lines denote 95% confidence interval bounds for *w*.

medRxiv preprint doi: https://doi.org/10.64898/2025.12.31.25343290; this version posted January 19, 2026. The copyright holder for this preprint
(which was not certified by peer review) is the author/funder, who has granted medRxiv a license to display the preprint in perpetuity.
It is made available under a CC-BY 4.0 International license .

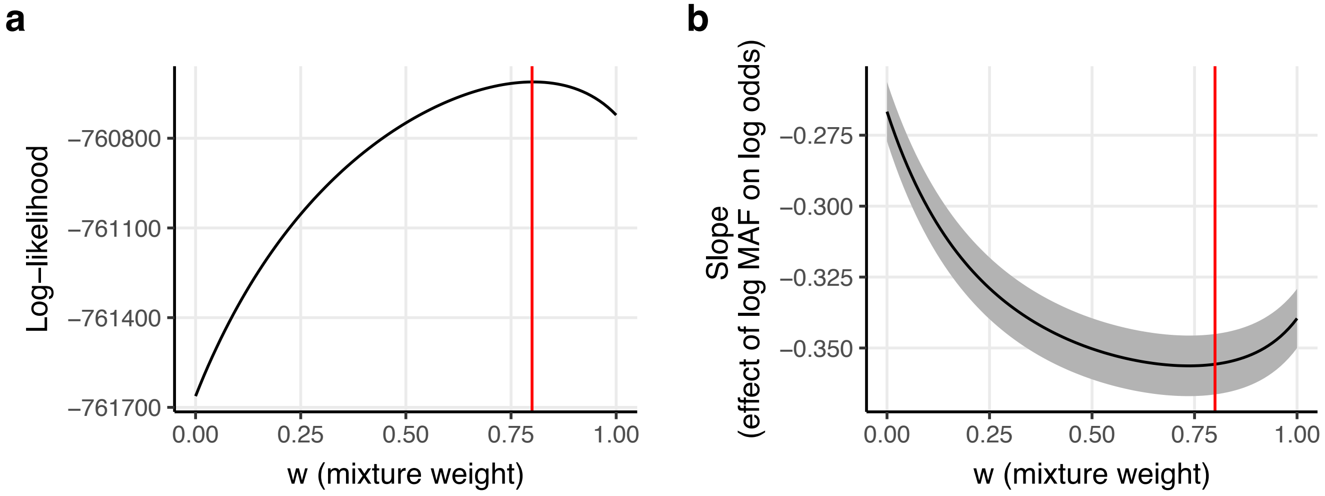

Supplementary Figure 9: Best-fit parameters for a logistic regression of non-synonymous status on log(p_{mix}), thresholding MAF at an alternate value of 0.05. We report **(a)** the profile log-likelihood with respect to *w*, treating the regression intercept and slope as nuisance parameters, thresholding p_A and p_E at 0.05, and **(b)** the regression slope (γ) for values of *w* between 0 and 1, thresholding p_A and p_E at 0.05. If we "threshold" a SNP at a MAF of *c*, it means that if a SNP has $p_A < c$, we set p_A to c, and if a SNP has $p_E < c$, we set p_E to c. In the main analyses, we threshold all MAF values at 0.002 (after filtering). Red lines in (a) and (b) denote the MLE estimate of *w*. The shaded region in (b) denotes 95% confidence intervals for γ.

medRxiv preprint doi: https://doi.org/10.64898/2025.12.31.25343290; this version posted January 19, 2026. The copyright holder for this preprint (which was not certified by peer review) is the author/funder, who has granted medRxiv a license to display the preprint in perpetuity.
It is made available under a CC-BY 4.0 International license .

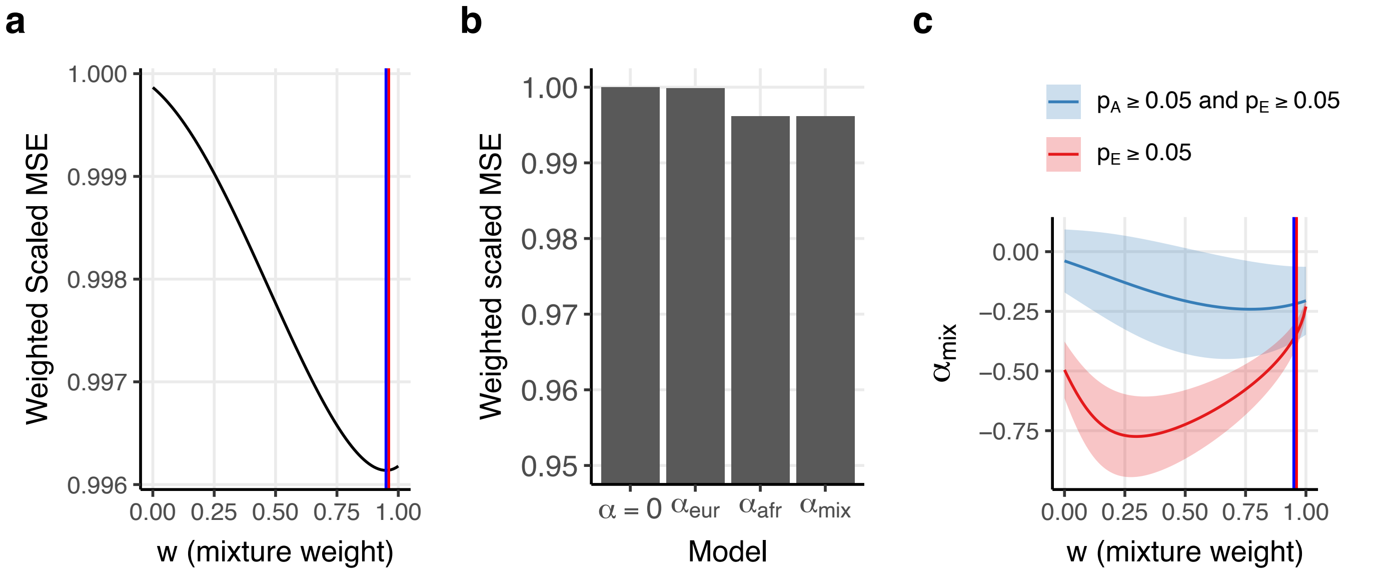

Supplementary Figure 10: Results of fitting the α_{mix} model across 50 diseases/traits restricted to variants with $p_A \geq 0.05$ and $p_E \geq 0.05$. We report **(a)** the weighted scaled mean squared error with respect to w, **(b)** the weighted scaled mean squared error for various models, including a model without MAF dependence (α=0), a model with European MAF dependence (α_{eur}), a model with African MAF dependence (α_{afr}), and the α_{mix} model, and **(c)** estimates of α_{mix} as a function of w. Weighted scaled MSE weights the MSE for each bivariate MAF bin by the number of SNPs it contains and scales the sum over all traits such that the null model has a weighted scaled MSE of 1. Vertical lines in (a) and (c) denote point estimates of w. Blue lines correspond to estimates restricted to SNPs with $p_A \geq 0.05$ and $p_E \geq 0.05$ and red lines correspond to estimates from the main analyses (restricted to SNPs with $p_A \geq 0.05$). Shaded regions in (c) denote 95% confidence intervals.

medRxiv preprint doi: https://doi.org/10.64898/2025.12.31.25343290; this version posted January 19, 2026. The copyright holder for this preprint
(which was not certified by peer review) is the author/funder, who has granted medRxiv a license to display the preprint in perpetuity.
It is made available under a CC-BY 4.0 International license .

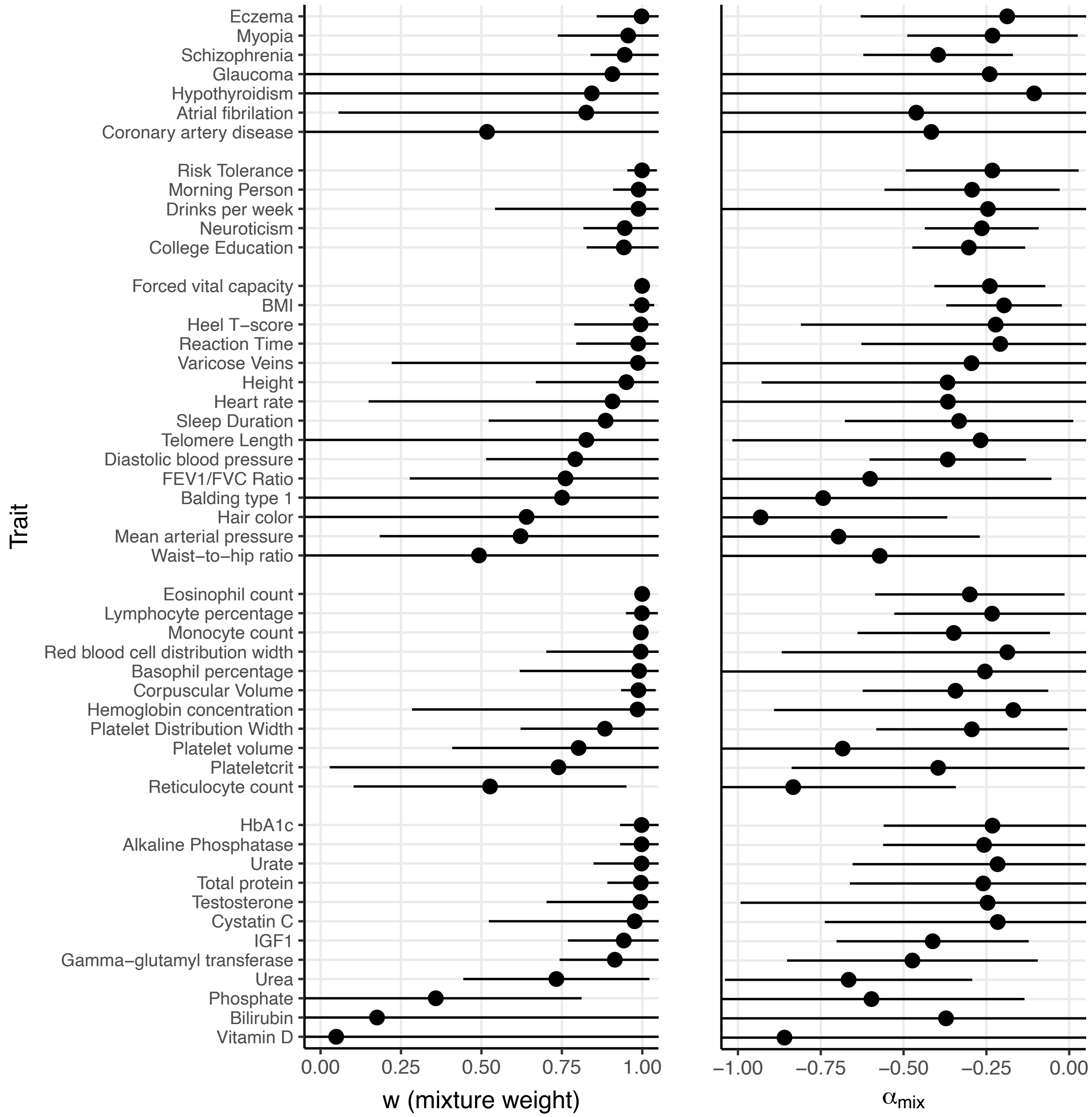

Supplementary Figure 11: Results of fitting the α_{mix} model separately for 50 diseases/traits. We report estimates of **(a)** *w* and **(b)** α_{mix}. Diseases/traits are ordered by point estimate of *w* within disease/trait categories. Error bars denote 95% confidence intervals. Numerical results for all 50 traits are reported in Supplementary Table 11.

medRxiv preprint doi: https://doi.org/10.64898/2025.12.31.25343290; this version posted January 19, 2026. The copyright holder for this preprint (which was not certified by peer review) is the author/funder, who has granted medRxiv a license to display the preprint in perpetuity.
It is made available under a CC-BY 4.0 International license .

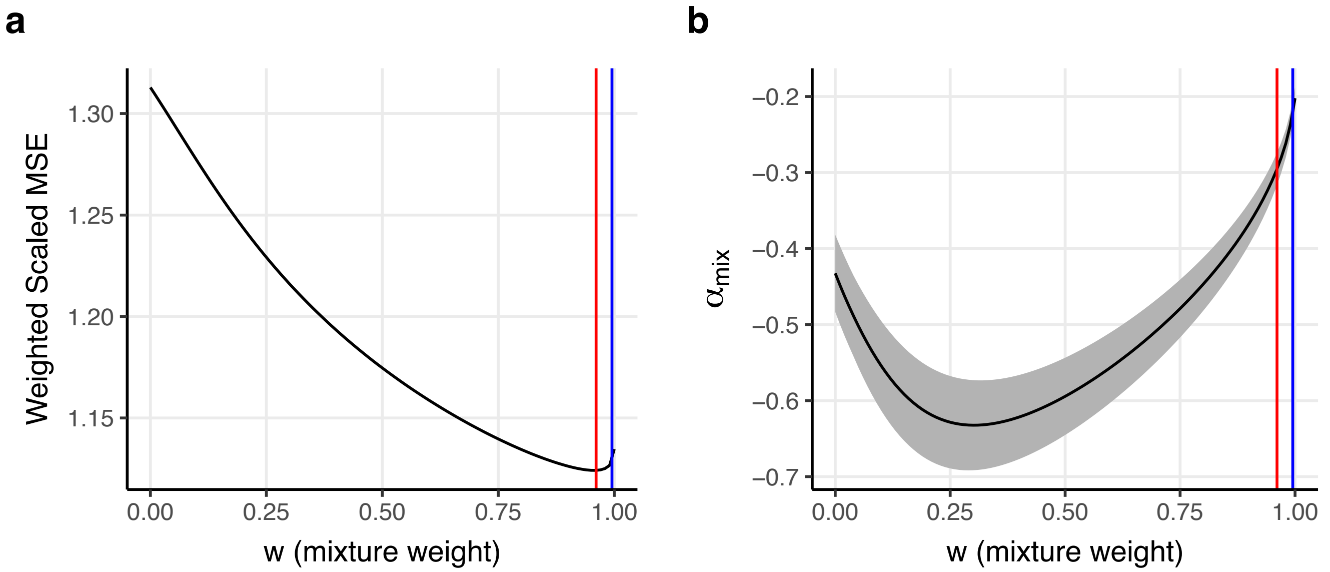

Supplementary Figure 12: **Results of fitting the α_{mix} model across 50 diseases/traits via a random effects meta-analysis of disease/trait-specific alpha-mix parameters.** We report **(a)** the weighted scaled mean squared error, summed across traits, with respect to *w*, and **(b)** estimates of α_{mix} as a function of *w*. Blue lines denote our meta-analyzed point estimate of *w*. Red lines denote the point estimate of *w* from the main analysis. The shaded region in (b) denotes 95% confidence intervals for α_{mix}. Weighted scaled MSE was scaled by an arbitrary constant. The random effects meta-analysis estimate of *w* is not guaranteed to minimize the MSE because *w* is estimated separately for each trait, and some traits have MSE functions which are minimized at values other than the minimum of the shared MSE.

medRxiv preprint doi: https://doi.org/10.64898/2025.12.31.25343290; this version posted January 19, 2026. The copyright holder for this preprint (which was not certified by peer review) is the author/funder, who has granted medRxiv a license to display the preprint in perpetuity.
It is made available under a CC-BY 4.0 International license .

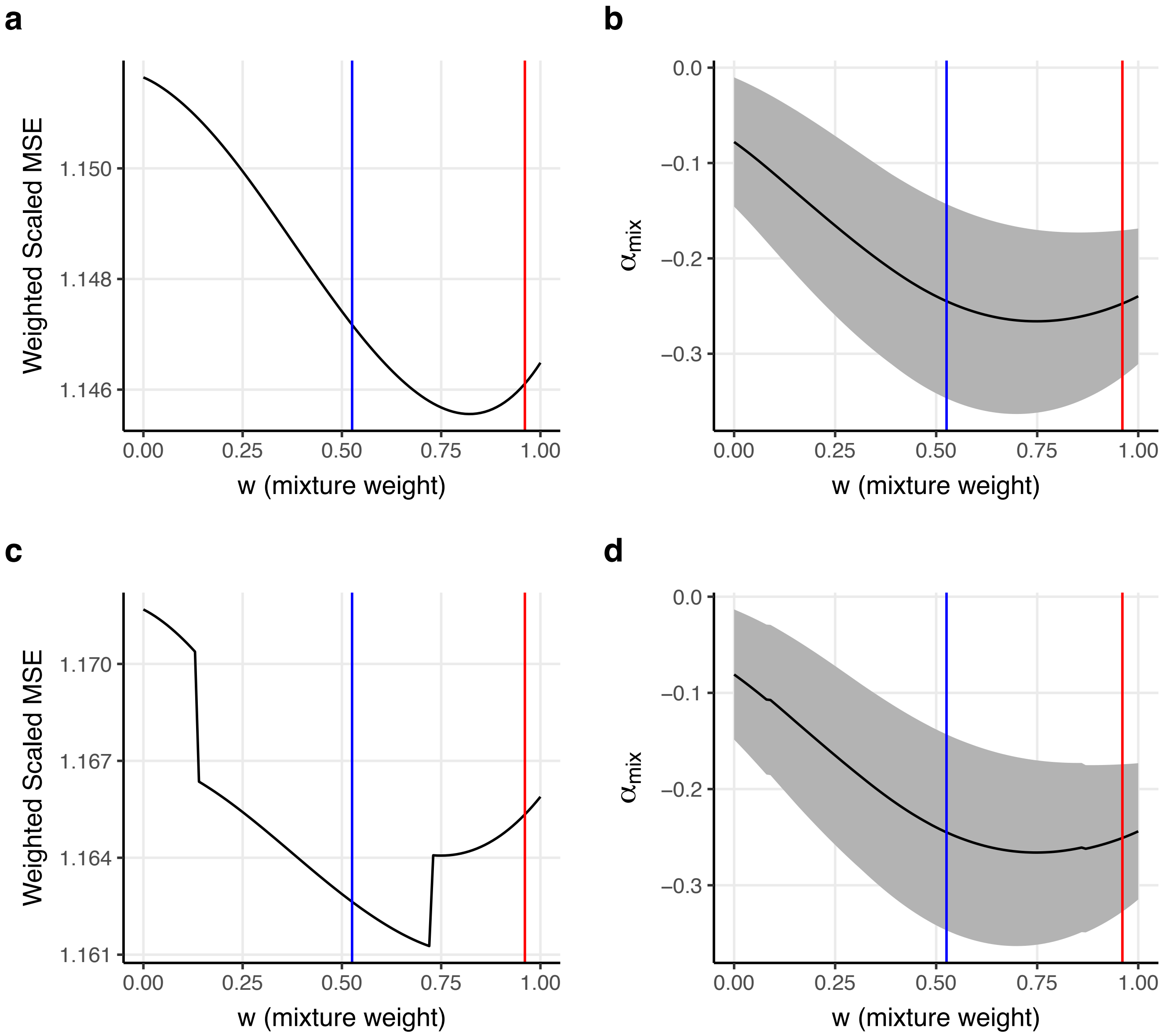

Supplementary Figure 13: **Results of fitting the α_{mix} model across 50 diseases/traits via a random effects meta-analysis of disease/trait-specific alpha-mix parameters restricted to SNPs with $p_A \geq 0.05$ and $p_E \geq 0.05$.** We report **(a)** the weighted scaled mean squared error with respect to *w*, summed across diseases/traits while restricting to SNPs with $p_A \geq 0.05$ and $p_E \geq 0.05$, excluding UKB_460K.biochemistry_Phosphate, **(b)** α_{mix} as a function of *w*, estimated using a random effects meta-analysis across traits while restricting to SNPs with $p_A \geq 0.05$ and $p_E \geq 0.05$ excluding UKB_460K.biochemistry_Phosphate, **(c)** same as (a), including UKB_460K.biochemistry_Phosphate, and **(d)** same as (b), including UKB_460K.biochemistry_Phosphate. Blue lines denote our meta-analyzed point estimate of *w*. Red lines denote the point estimate of *w* from the main analysis. The shaded regions in (b,d) denote 95% confidence intervals for α_{mix}.

The random effects meta-analysis estimate of *w* is not guaranteed to minimize the MSE because *w* is estimated separately for each trait, and some traits have MSE functions which are minimized at values other than the minimum of the shared MSE function.

Inclusion of UKB_460K.biochemistry_Phosphate had very little effect on our meta-analyzed estimates of *w* (difference of .0052) and α_{mix} (difference of .0040).

Weighted scaled MSE was scaled by an arbitrary constant.

medRxiv preprint doi: https://doi.org/10.64898/2025.12.31.25343290; this version posted January 19, 2026. The copyright holder for this preprint (which was not certified by peer review) is the author/funder, who has granted medRxiv a license to display the preprint in perpetuity.
It is made available under a CC-BY 4.0 International license .

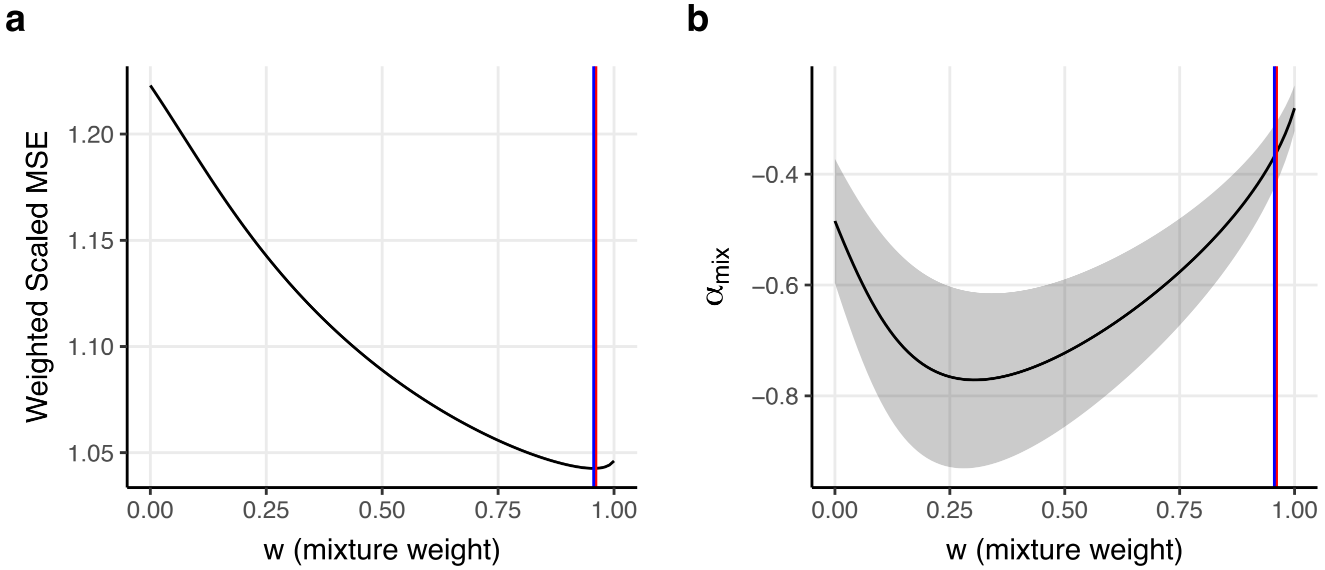

Supplementary Figure 14: Results of fitting the α_{mix} model across 50 diseases/traits while estimating African MAF using *N*=107 Yoruban genomes. We report **(a)** the weighted scaled mean squared error with respect to *w*, and **(b)** estimates of α_{mix} as a function of *w*. Blue lines denote our point estimate of *w* while estimating African MAF using *N*=107 Yoruban genomes. Red lines denote the point estimate of *w* from the main analysis. The shaded region in (b) denotes 95% confidence intervals for α_{mix}. Yoruban genomes were used to define the 2-dimensional MAF bins which are used both while estimating per-allele effect size variance using S-LDSC and while estimating the parameters of the α_{mix} model.
Weighted scaled MSE was scaled by an arbitrary constant.

medRxiv preprint doi: https://doi.org/10.64898/2025.12.31.25343290; this version posted January 19, 2026. The copyright holder for this preprint (which was not certified by peer review) is the author/funder, who has granted medRxiv a license to display the preprint in perpetuity.
It is made available under a CC-BY 4.0 International license .

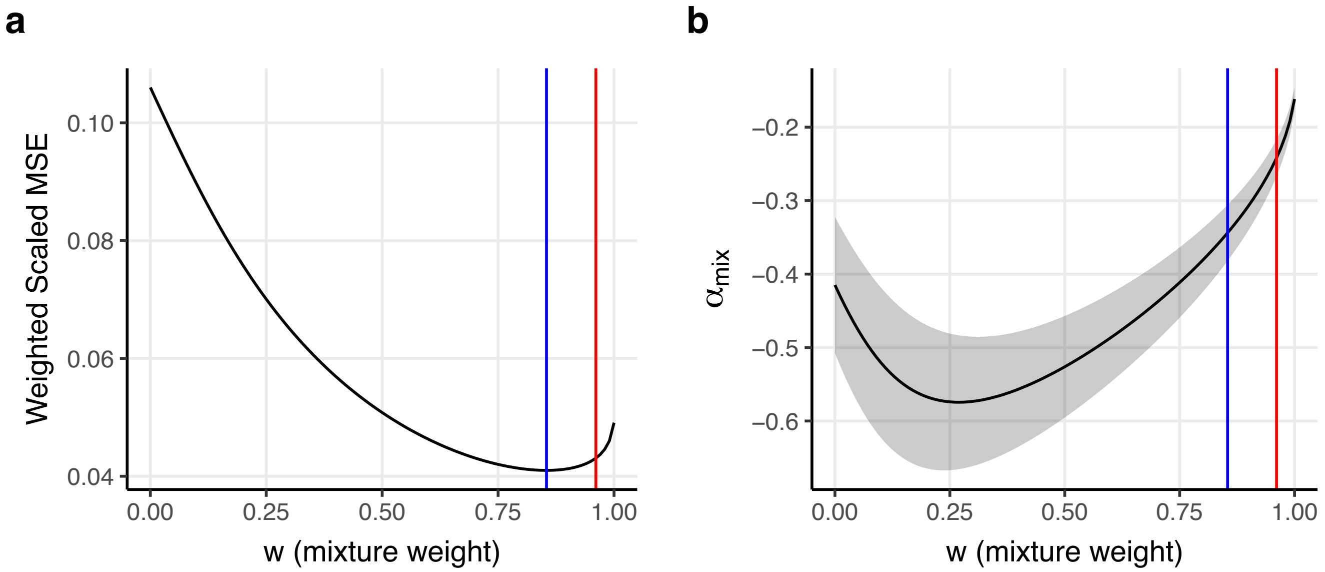

Supplementary Figure 15: Results of fitting the α_{mix} model across 50 diseases/traits using the standard Baseline-LD (v2.2) model without annotations for African MAF. We report **(a)** the weighted scaled mean squared error with respect to *w*, and **(b)** estimates of α_{mix} as a function of *w*. Blue lines denote our point estimate of *w* using the standard Baseline-LD (v2.2) model without annotations for African MAF. Red lines denote the point estimate of *w* from the main analysis. The shaded region in (b) denotes 95% confidence intervals for α_{mix}. The European MAF bins included in the standard Baseline-LD (v2.2) model were retained.
Weighted scaled MSE was scaled by an arbitrary constant.

medRxiv preprint doi: https://doi.org/10.64898/2025.12.31.25343290; this version posted January 19, 2026. The copyright holder for this preprint
(which was not certified by peer review) is the author/funder, who has granted medRxiv a license to display the preprint in perpetuity.
It is made available under a CC-BY 4.0 International license .

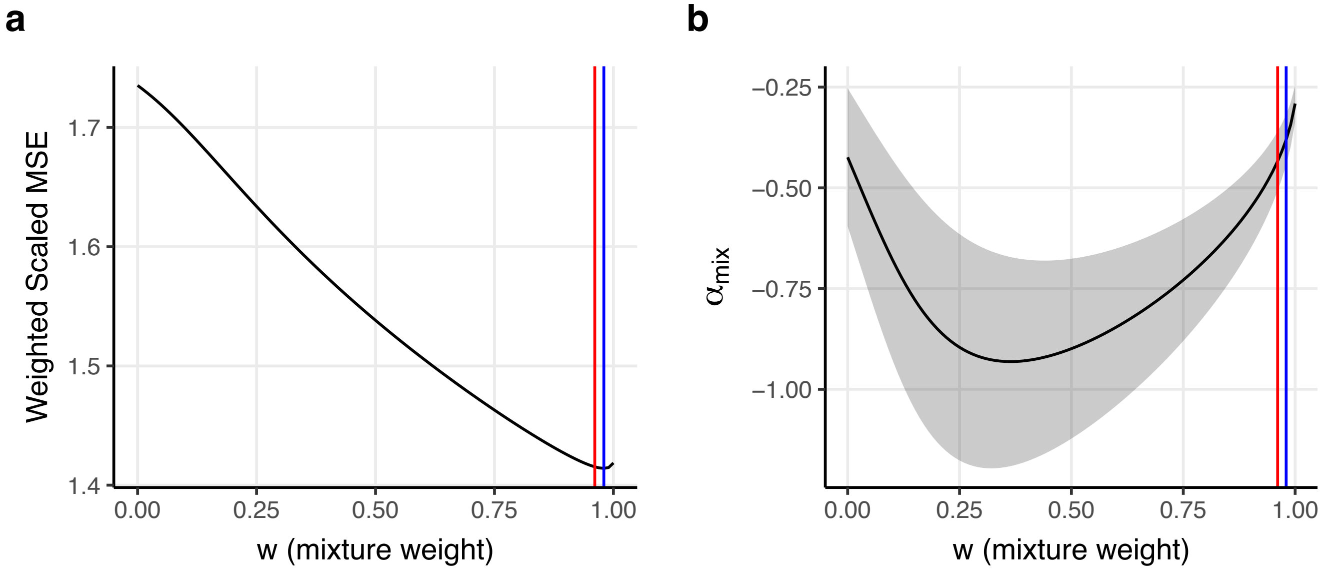

Supplementary Figure 16: Results of fitting the α_{mix} model across 50 diseases/traits using 2-dimensional MAF bin annotations without including the BaselineLD-v2.2 model. We report **(a)** the weighted scaled mean squared error with respect to *w*, and **(b)** estimates of α_{mix} as a function of *w*. Blue lines denote our point estimate of *w* using 2-dimensional MAF bin annotations without including the BaselineLD-v2.2 model. Red lines denote the point estimate of *w* from the main analysis. The shaded region in (b) denotes 95% confidence intervals for α_{mix}. Weighted scaled MSE was scaled by an arbitrary constant.

medRxiv preprint doi: https://doi.org/10.64898/2025.12.31.25343290; this version posted January 19, 2026. The copyright holder for this preprint (which was not certified by peer review) is the author/funder, who has granted medRxiv a license to display the preprint in perpetuity.
It is made available under a CC-BY 4.0 International license .

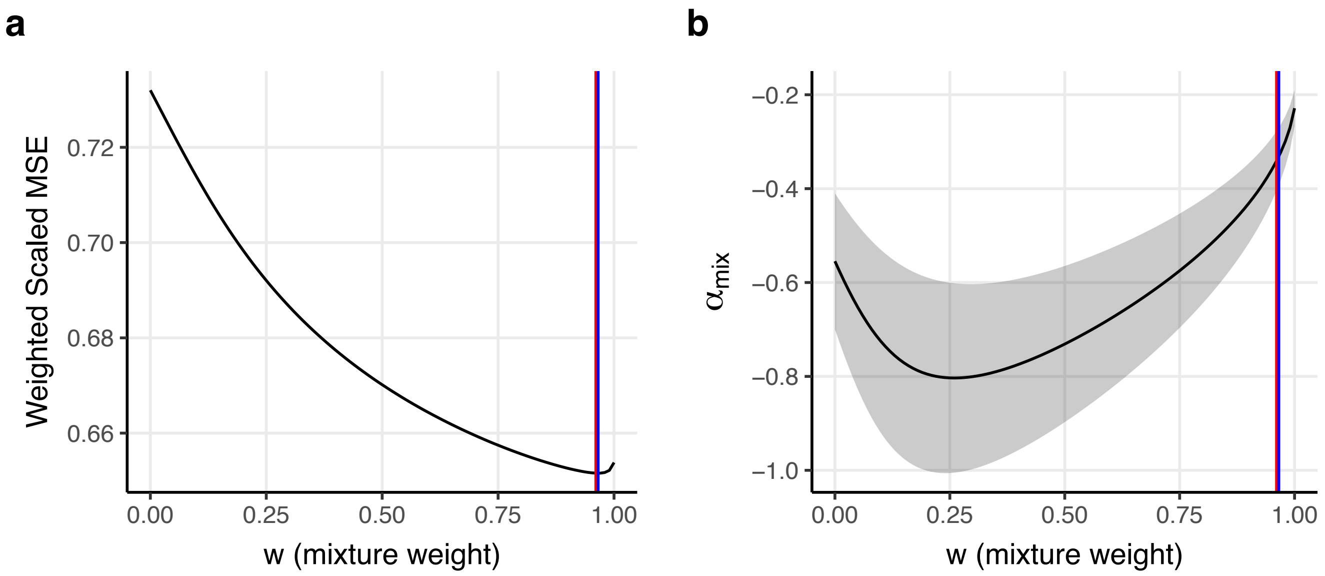

Supplementary Figure 17: Results of fitting the α_{mix} model across 29 diseases/traits from UK Biobank. We report **(a)** the weighted scaled mean squared error with respect to *w*, and **(b)** estimates of α_{mix} as a function of *w*. Blue lines denote our point estimate of *w* using 29 UK Biobank diseases/traits. Red lines denote the point estimate of *w* from the main analysis. The shaded region in (b) denotes 95% confidence intervals for α_{mix}.
Weighted scaled MSE was scaled by an arbitrary constant.

medRxiv preprint doi: https://doi.org/10.64898/2025.12.31.25343290; this version posted January 19, 2026. The copyright holder for this preprint (which was not certified by peer review) is the author/funder, who has granted medRxiv a license to display the preprint in perpetuity.
It is made available under a CC-BY 4.0 International license .

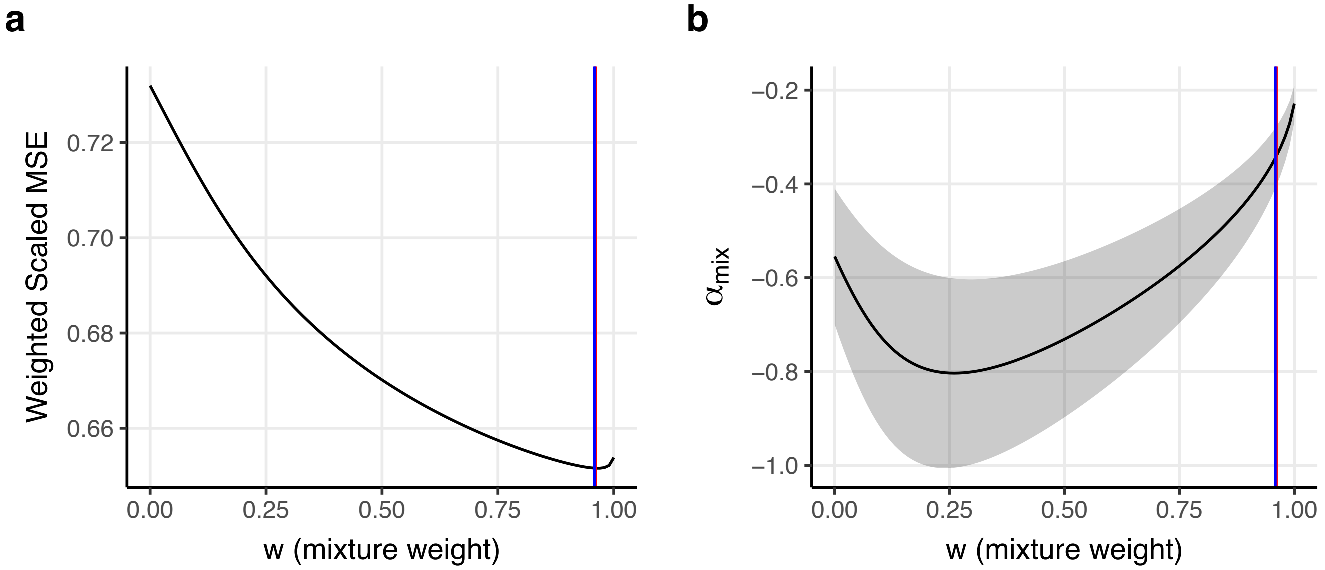

Supplementary Figure 18: Results of fitting the α_{mix} model across 21 diseases/traits from publicly available summary statistics. We report **(a)** the weighted scaled mean squared error with respect to *w*, and **(b)** estimates of α_{mix} as a function of *w*. Blue lines denote our point estimate of *w* using 21 diseases/traits from publicly available summary statistics (not including any diseases/traits from UK Biobank). Red lines denote the point estimate of *w* from the main analysis. The shaded region in (b) denotes 95% confidence intervals for α_{mix}. Weighted scaled MSE was scaled by an arbitrary constant.

medRxiv preprint doi: https://doi.org/10.64898/2025.12.31.25343290; this version posted January 19, 2026. The copyright holder for this preprint (which was not certified by peer review) is the author/funder, who has granted medRxiv a license to display the preprint in perpetuity.
It is made available under a CC-BY 4.0 International license .

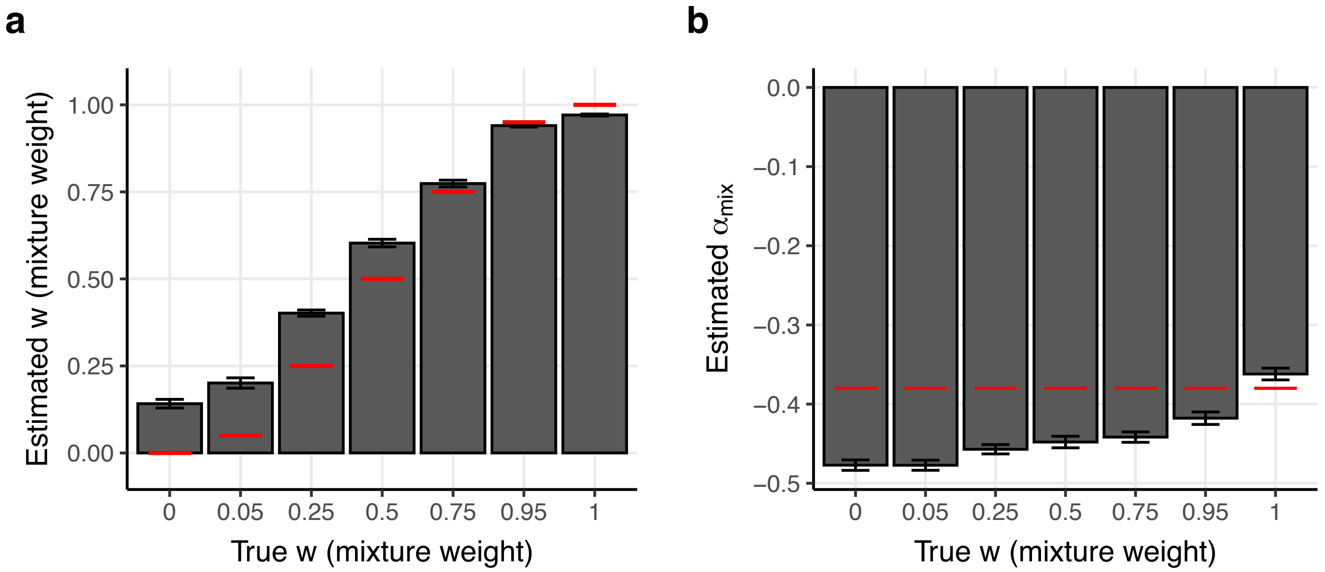

Supplementary Figure 19: Results of fitting the α_{mix} model across groups of 25 traits in simulations, while using European MAF from UK Biobank for the generative model, and European MAF from 1000 Genomes for inference. We report estimates of w **(a)** and α_{mix} **(b)**. Bars denote the mean estimate of each parameter across 40 replicates. Red lines denote the true value of each parameter. Error bars denote 95% confidence intervals. 1000 Genomes MAF was used both to define MAF bin annotations given to S-LDSC and during α_{mix} inference.

medRxiv preprint doi: https://doi.org/10.64898/2025.12.31.25343290; this version posted January 19, 2026. The copyright holder for this preprint (which was not certified by peer review) is the author/funder, who has granted medRxiv a license to display the preprint in perpetuity.
It is made available under a CC-BY 4.0 International license .

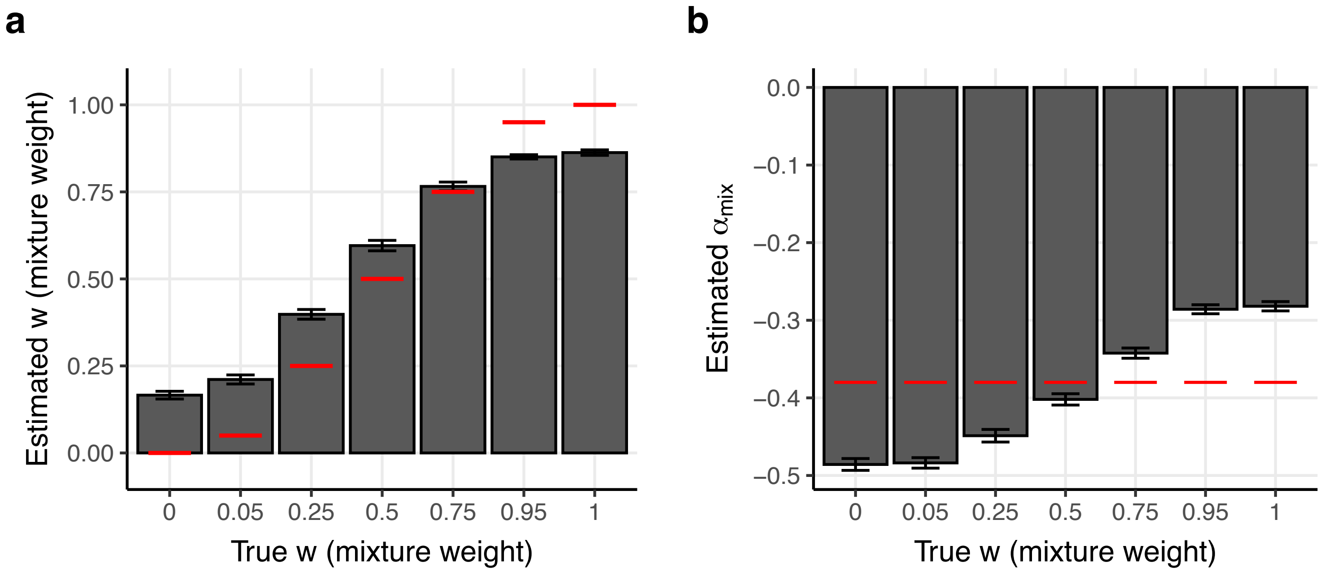

Supplementary Figure 20: Results of fitting the α_{mix} model across groups of 25 traits in simulations, while thresholding MAF at *T*=0.05. We report estimates of w **(a)** and α_{mix} **(b)**. Bars denote the mean estimate of each parameter across 40 replicates. Red lines denote the true value of each parameter. Error bars denote 95% confidence intervals. *T* is only used by the generative model, not by S-LDSC or in α_{mix} inference.

medRxiv preprint doi: https://doi.org/10.64898/2025.12.31.25343290; this version posted January 19, 2026. The copyright holder for this preprint (which was not certified by peer review) is the author/funder, who has granted medRxiv a license to display the preprint in perpetuity.
It is made available under a CC-BY 4.0 International license .

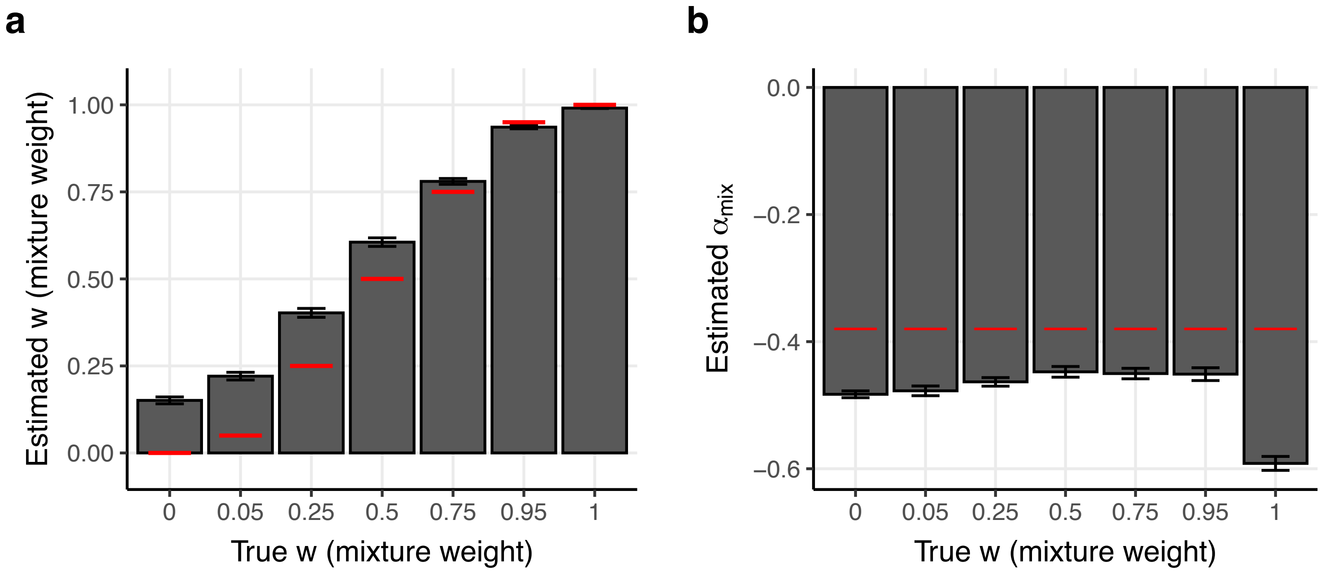

Supplementary Figure 21: Results of fitting the α_{mix} model across groups of 25 traits in simulations, while thresholding MAF at T=1.5*10^{-5} (equivalent to a minor allele count of 1 in our sample of genomes with local African ancestry from All of Us). We report estimates of w **(a)** and α_{mix} **(b)**. Bars denote the mean estimate of each parameter across 40 replicates. Red lines denote the true value of each parameter. Error bars denote 95% confidence intervals. T is only used by the generative model, not by S-LDSC or in α_{mix} inference.

medRxiv preprint doi: https://doi.org/10.64898/2025.12.31.25343290; this version posted January 19, 2026. The copyright holder for this preprint (which was not certified by peer review) is the author/funder, who has granted medRxiv a license to display the preprint in perpetuity.
It is made available under a CC-BY 4.0 International license .

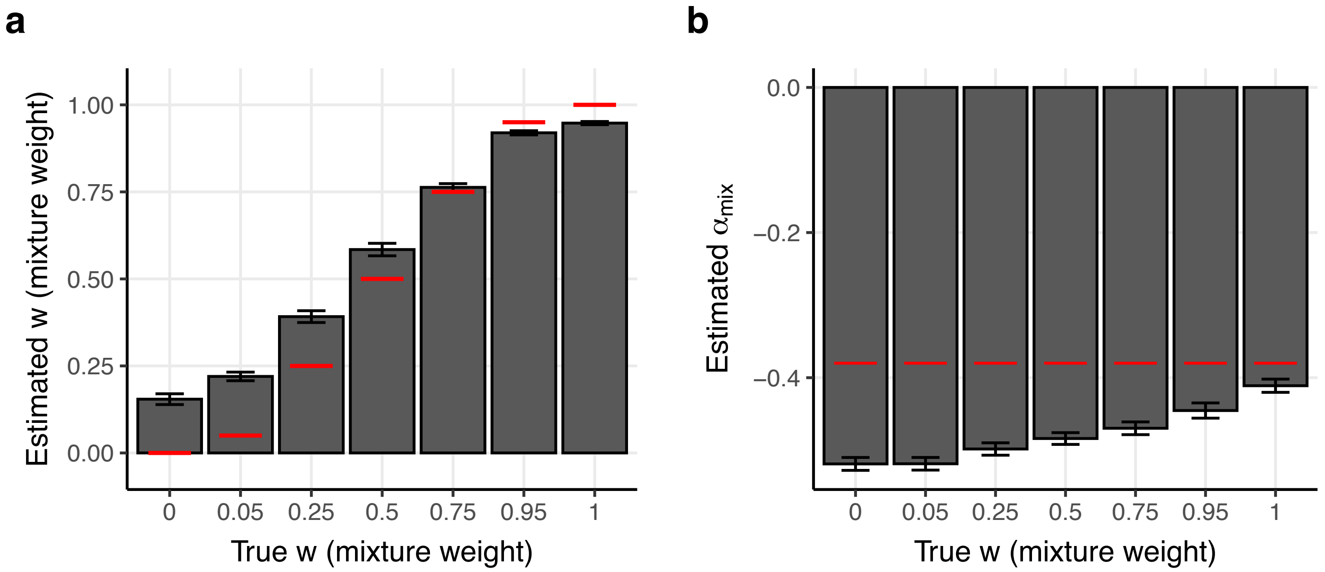

Supplementary Figure 22: Results of fitting the α_{mix} model across groups of 25 traits in simulations for traits with 5,000 causal variants. We report estimates of w **(a)** and α_{mix} **(b)**. Bars denote the mean estimate of each parameter across 40 replicates. Red lines denote the true value of each parameter. Error bars denote 95% confidence intervals.

medRxiv preprint doi: https://doi.org/10.64898/2025.12.31.25343290; this version posted January 19, 2026. The copyright holder for this preprint (which was not certified by peer review) is the author/funder, who has granted medRxiv a license to display the preprint in perpetuity.
It is made available under a CC-BY 4.0 International license .

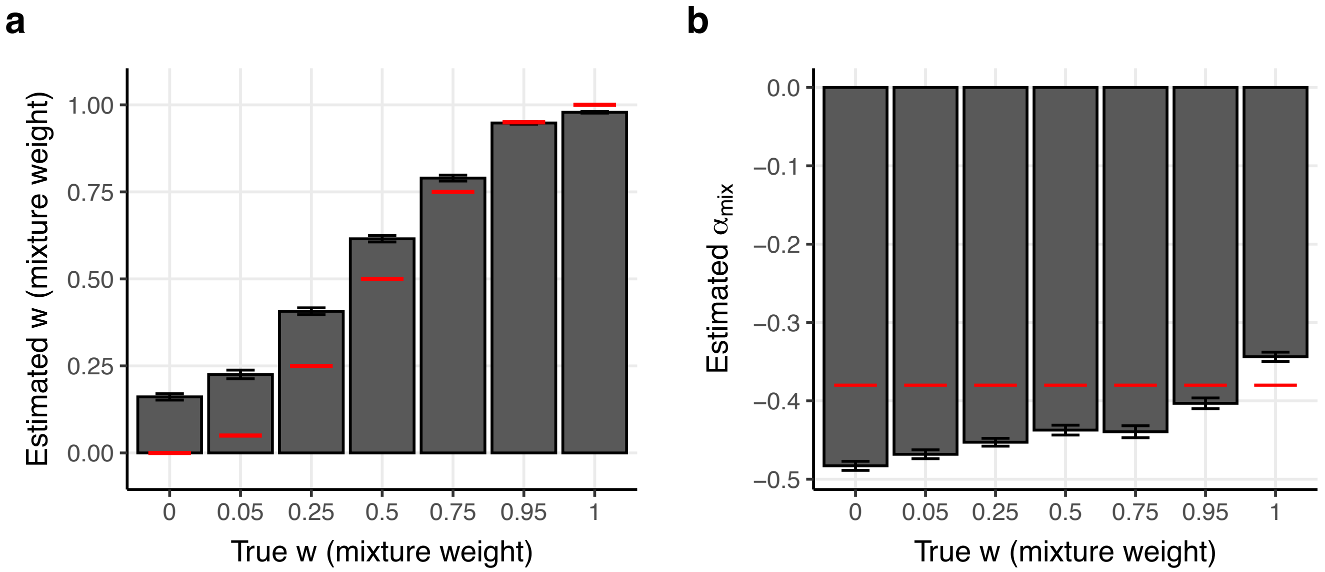

Supplementary Figure 23: Results of fitting the α_{mix} model across groups of 25 traits in simulations for traits with 20,000 causal variants. We report estimates of w **(a)** and α_{mix} **(b)**. Bars denote the mean estimate of each parameter across 40 replicates. Red lines denote the true value of each parameter. Error bars denote 95% confidence intervals.

medRxiv preprint doi: https://doi.org/10.64898/2025.12.31.25343290; this version posted January 19, 2026. The copyright holder for this preprint (which was not certified by peer review) is the author/funder, who has granted medRxiv a license to display the preprint in perpetuity.
It is made available under a CC-BY 4.0 International license .

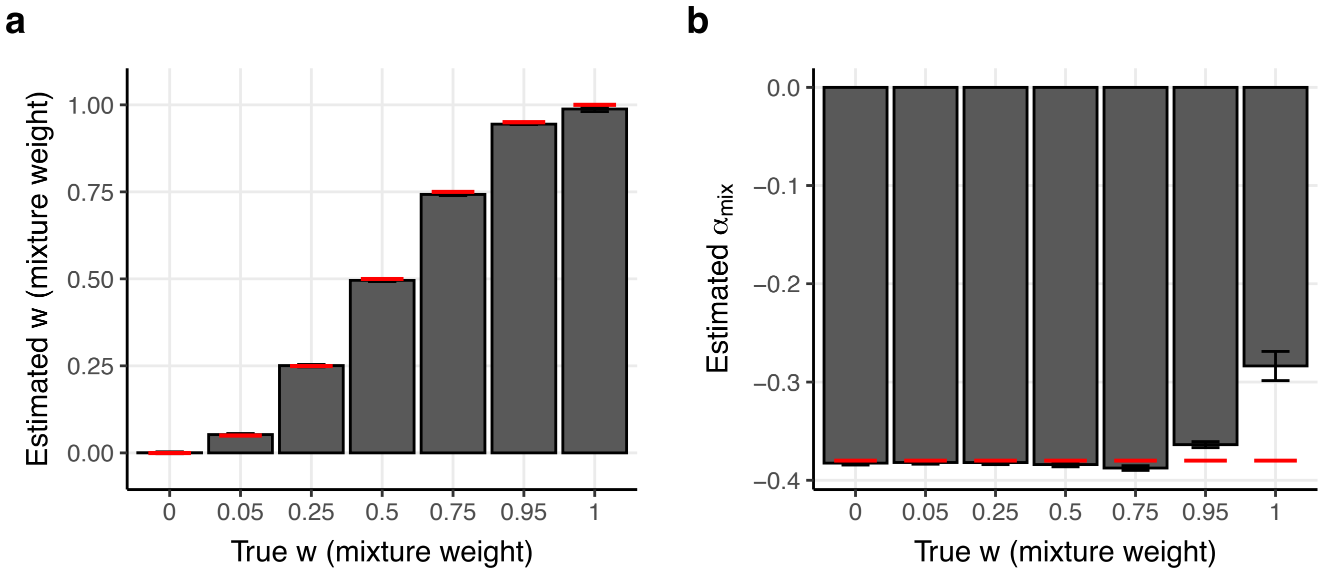

Supplementary Figure 24: Results of fitting the α_{mix} model across groups of 25 traits in simulations using exact values of β^2 during inference, rather than estimates from S-LDSC. We report estimates of w **(a)** and α_{mix} **(b)**. Bars denote the mean estimate of each parameter across 40 replicates. Red lines denote the true value of each parameter. Error bars denote 95% confidence intervals.

medRxiv preprint doi: https://doi.org/10.64898/2025.12.31.25343290; this version posted January 19, 2026. The copyright holder for this preprint (which was not certified by peer review) is the author/funder, who has granted medRxiv a license to display the preprint in perpetuity.
It is made available under a CC-BY 4.0 International license .

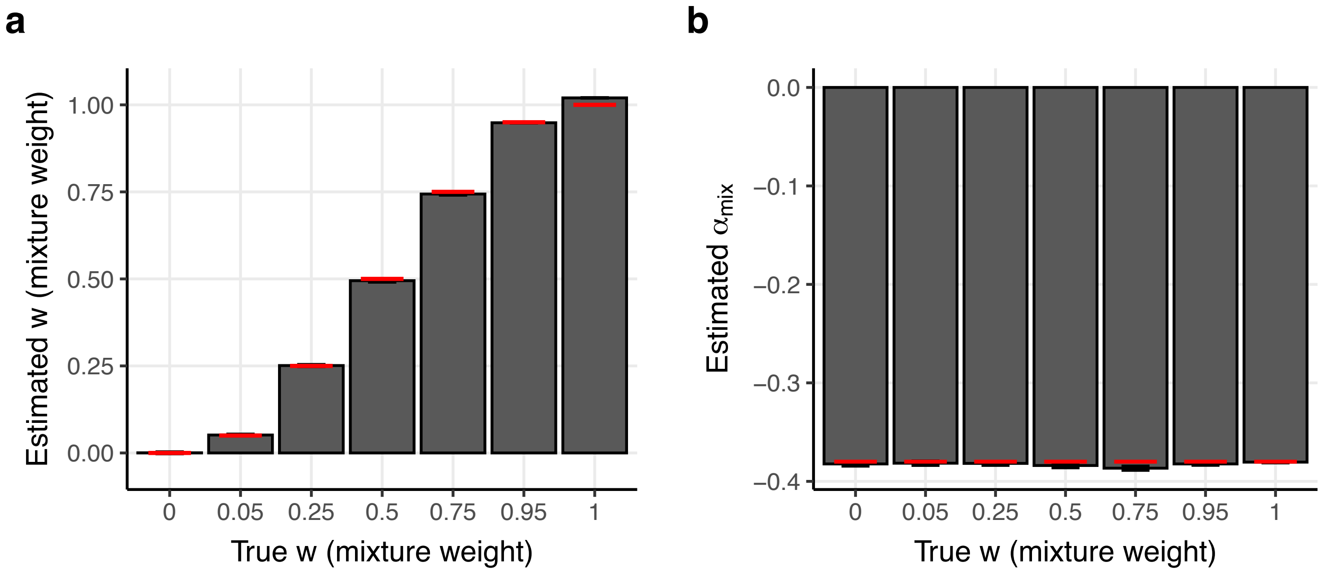

Supplementary Figure 25: Results of fitting the α_{mix} model across groups of 25 traits in simulations while using exact values of β^2 (rather than estimates from S-LDSC), values of p_A and p_E derived from causal variants (rather than all variants), and thresholding p_{mix} at T=0.005. We report estimates of w **(a)** and α_{mix} **(b)**. Bars denote the mean estimate of each parameter across 40 replicates. Red lines denote the true value of each parameter. Error bars denote 95% confidence intervals. p_{mix} was computed separately for each causal SNP, thresholded at T=0.005, and then averaged over all causal SNPs in each two-dimensional MAF bin.

medRxiv preprint doi: https://doi.org/10.64898/2025.12.31.25343290; this version posted January 19, 2026. The copyright holder for this preprint (which was not certified by peer review) is the author/funder, who has granted medRxiv a license to display the preprint in perpetuity.
It is made available under a CC-BY 4.0 International license .

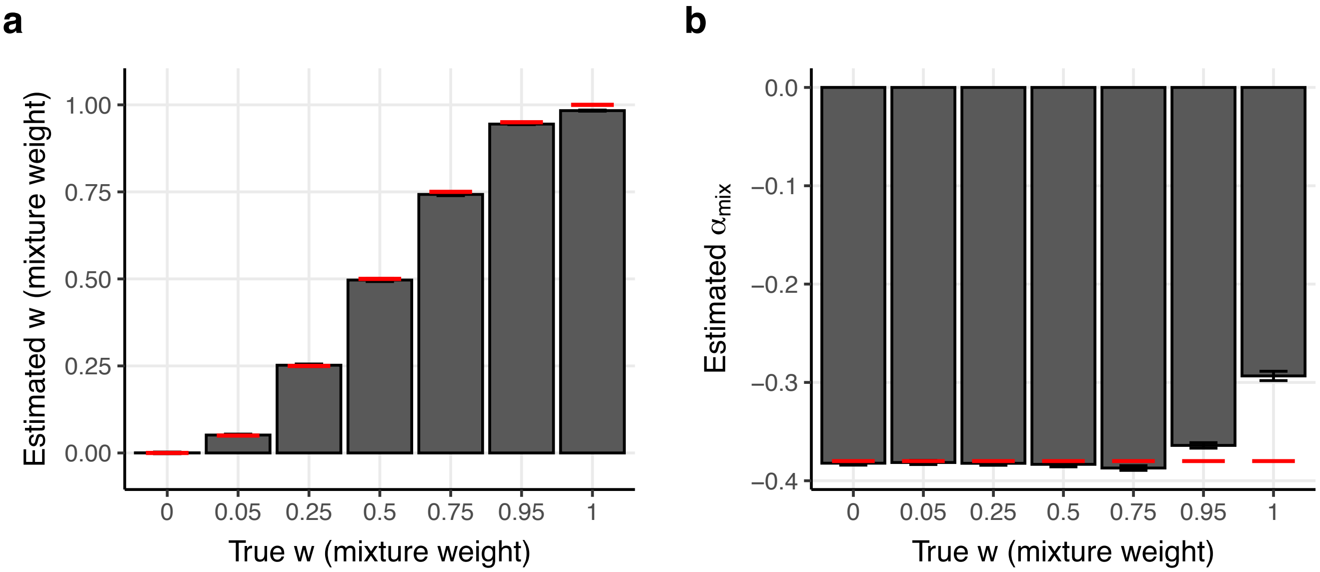

Supplementary Figure 26: Results of fitting the α_{mix} model across groups of 25 traits in simulations while using exact values of β^2 (rather than estimates from S-LDSC) and values of p_A and p_E derived from causal variants (rather than all variants). We report estimates of *w* **(a)** and α_{mix} **(b)**. Bars denote the mean estimate of each parameter across 40 replicates. Red lines denote the true value of each parameter. Error bars denote 95% confidence intervals. p_{mix} was computed separately for each causal SNP, thresholded at $1.5*10^{-5}$ (equivalent to a minor allele count of 1 in our sample of genomes with local African ancestry from All of Us), and then averaged over all causal SNPs in each two-dimensional MAF bin.

medRxiv preprint doi: https://doi.org/10.64898/2025.12.31.25343290; this version posted January 19, 2026. The copyright holder for this preprint (which was not certified by peer review) is the author/funder, who has granted medRxiv a license to display the preprint in perpetuity.
It is made available under a CC-BY 4.0 International license .

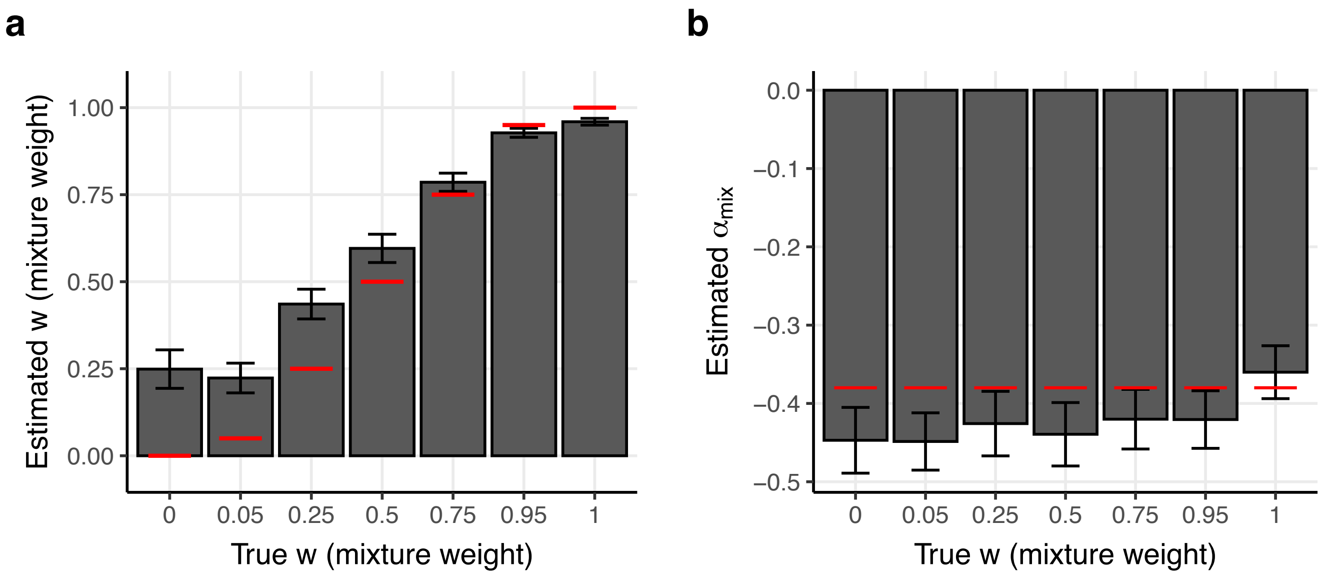

Supplementary Figure 27: Results of fitting the α_{mix} model separately for each trait in simulations. We report estimates of w **(a)** and α_{mix} **(b)**. Bars denote the mean estimate of each parameter across 100 replicates (each with a single trait). Red lines denote the true value of each parameter. Error bars denote 95% confidence intervals.
